# Supplementary material for: ITGAM is a risk factor to systemic lupus erythematosus and possibly a protection factor to rheumatoid arthritis in patients from Mexico
Source: PLoS One. 2019 Nov 27;14(11):e0224543. doi: 10.1371/journal.pone.0224543 (PMC6881022; doi:10.1371/journal.pone.0224543)
Supplement: S1 Table — (DOCX) [file pone.0224543.s001.docx]

**S1 Table.** Primer sequences were used to amplify two SNPs.

| ID | Sequence 5'-3' | bp |
| --- | --- | --- |
| rs1143683- ITGAM-F | TGAGGACTCCTACAGGACACA | 314 |
| rs1143683- ITGAM-R | GAGGAGCCAGGAGTTCTGACC |  |
| rs1143679- ITGAM-F | AGGAAGACCTTTTCTCATTCTGAGT | 523 |
| rs1143679- ITGAM-R | GACAAGGAGGTCTGACGGTG |  |
