# Supplementary material for: ITGAM is a risk factor to systemic lupus erythematosus and possibly a protection factor to rheumatoid arthritis in patients from Mexico
Source: PLoS One. 2019 Nov 27;14(11):e0224543. doi: 10.1371/journal.pone.0224543 (PMC6881022; doi:10.1371/journal.pone.0224543)
Supplement: S2 Table — (DOCX) [file pone.0224543.s002.docx]

**S2 Table**. Meta-analysis of four ITGAM SNPs with SLE across two cohorts.

| SNP | A1/A2 | Z-score | p-val | Direction  of ORs | P-het |
| --- | --- | --- | --- | --- | --- |
| rs34572943 | A/G | 5.098 | 3.43E-07 | ++ | 0.001 |
| rs1143679 | A/G | 4.452 | 8.50E-06 | ++ | 0.005 |
| rs9888739 | T/C | 4.538 | 5.69E-06 | ++ | 0.012 |
| rs1143683 | T/C | 4.593 | 4.36E-06 | ++ | 0.025 |

A1: risk allele, A2: non-risk allele. P_het: p-value for the heterogeneity test.
