# Supplementary material for: ITGAM is a risk factor to systemic lupus erythematosus and possibly a protection factor to rheumatoid arthritis in patients from Mexico
Source: PLoS One. 2019 Nov 27;14(11):e0224543. doi: 10.1371/journal.pone.0224543 (PMC6881022; doi:10.1371/journal.pone.0224543)
Supplement: S3 Table — (DOCX) [file pone.0224543.s003.docx]

**S3 Table.** Comparison of SNP alleles between TaqMan and Sanger sequencing.

|  | **fid** | **iid** | **Pheno*** | **rs1143679** | | **rs1143683** | |
| --- | --- | --- | --- | --- | --- | --- | --- |
|  |  |  |  | **TaqMan** | **Sanger** | **TaqMan** | **Sanger** |
| **1** | 118 | 118YUC | 2 | GG | GG | 00 | TC |
| **2** | 17CAD | 17CADYUC | 1 | AG | AG | TT | TT |
| **3** | 49 | 49YUC | 2 | 00 | AG | TC | TC |
| **4** | 11 | 11YUC | 2 | AG | AG | TC | TC |
| **5** | 32 | 32YUC | 2 | AG | AG | TC | TC |
| **6** | 35 | 35YUC | 2 | AG | AG | TC | TC |
| **7** | 36 | 36YUC | 2 | AG | AG | TC | TC |
| **8** | 43 | 43YUC | 2 | AG | AG | TC | TC |
| **9** | 77 | 77YUC | 2 | AG | AG | TC | TC |
| **10** | 80 | 80YUC | 2 | AG | AG | TC | TC |
| **11** | 100 | 100YUC | 2 | AG | AG | TC | TC |
| **12** | 131 | 131YUC | 2 | GG | GG | TC | TC |
| **13** | 189CAD | 189CADYUC | 1 | GG | GG | TC | TC |
| **14** | 143 | 143YUC | 2 | AG | AG | CC | CC |
| **15** | 135 | 135YUC | 2 | GG | GG | CC | CC |
| **16** | 153 | 153YUC | 2 | GG | GG | CC | CC |
| **17** | 159 | 159YUC | 2 | GG | GG | CC | CC |
| **18** | 42 | 42YUC | 2 | AA | AA | TT | TT |
| **19** | 115 | 115YUC | 2 | AA | AA | TC | TC |
| **20** | plate3-D12 | plate3-D12 | -9 | GG | GG | 00 | TC |
| **21** | Plate2-B12 | Plate2-B12 | -9 | GG | GG | CC | CC |

*1=Normal, 2=Affected
