# Supplementary material for: ITGAM is a risk factor to systemic lupus erythematosus and possibly a protection factor to rheumatoid arthritis in patients from Mexico
Source: PLoS One. 2019 Nov 27;14(11):e0224543. doi: 10.1371/journal.pone.0224543 (PMC6881022; doi:10.1371/journal.pone.0224543)
Supplement: Attachment — Submitted filename: Response_to_Reviewers_10_9_19.docx [file pone.0224543.s004.docx]

**Response to the Reviewers comments.**

The work by Ramirez-Bello and colleagues evaluates the well-established genetic association between ITGAM and lupus in two cohorts from Mexico. They replicate this genetic association in two Mexican populations and provide results suggesting a possible protective effect of ITGAM lupus-associated haplotype against RA. This is straight-forward work, well written, and replication of this genetic association in Mexican populations is worth reporting.

The authors need to address the following points before this can be accepted for publication:

Thank you for reviewing our manuscript, and here are our point-by-point responses to the comments.

**Q1)** As the authors are well aware, the single most important limitation in this work is the lack of ability, by study design, to assess and correct for population stratification. This is major issue in admixed populations such as in Mexico. This limitation although mentioned briefly in the manuscript, should be emphasized and also mentioned in the abstract. On the positive side, the authors did study patients and controls matched by region (central Mexico versus Yucatan).

**Response:** Yes, we agree with the reviewer regarding limitation of this study. As reviewer appreciated that to minimize the effect of population stratification, we have used the ethnicity-matched cases and controls from the Central Mexico and Yucatan. Now we have mentioned this in abstract and in the text.

**Q2)** The association between ITGAM and lupus in the Yucatan population is likely inflated as a result of low power and small sample size. This should be also noted in the abstract. Power has been estimated, and in particular given low power in the Yucatan cohort it is important to attempt to correct the odds ratio for inflation as a function of power similar to an approach used to calculate winner's curse effect.

**Response:** Yes, we agree that association results from Yucatan are likely to be inflated due to the small samples sizes, especially due to the low allele frequency, and now we have mentioned this in the abstract. As you suggested, we have performed a meta-analysis, which is adjusted by the samples sizes. As expected, the minor allele frequencies and ORs are in consistent with the directionality, although there is a significant heterogeneity of ORs. We have provided all this information in the Supplementary Table 1.

**Q3)** Please attempt to perform a meta-analysis combining both Yucatan and Central Mexico lupus cohort.

**Response:** Yes, we have performed a meta-analysis, and information is provided in the **Supplementary Table 3**.

4) Other minor points:

- Please amend the first sentence in the conclusion to say: "Our data show that all four ITGAM SNPs are significantly associated with susceptibility to SLE in patients from Mexico."

**Response:** Done, thank you.

- Table 1 legend should say "Central Mexico"

**Response:** Corrected, thank you.

- Table 3 RA findings are not from Yucatan but from Central Mexico.

**Response:** Corrected. Thank you.
